# The Happy Older Latinos are Active (HOLA) health promotion and prevention study: study protocol for a pilot randomized controlled trial

**DOI:** 10.1186/s13063-015-1113-3

**Published:** 2015-12-18

**Authors:** Daniel E. Jimenez, Charles F. Reynolds, Margarita Alegría, Philip Harvey, Stephen J. Bartels

**Affiliations:** Department of Psychiatry and Behavioral Sciences, University of Miami Miller School of Medicine, 1695 NW 9th Ave., Suite 3208, Miami, FL 33136 USA; NIMH Center for Late Life Depression Prevention and Treatment, University of Pittsburgh School of Medicine and Graduate School of Public Health, Pittsburgh, PA USA; Department of Psychiatry, Harvard Medical School, Cambridge, MA USA; Dartmouth Centers for Health and Aging, Geisel School of Medicine at Dartmouth, Lebanon, NH USA

**Keywords:** Latinos, Mental illness prevention, Older adults, Health promotion

## Abstract

**Background:**

Results of previous studies attest to the greater illness burden of common mental disorders (anxiety and depression) in older Latinos and the need for developing preventive interventions that are effective, acceptable, and scalable. Happy Older Latinos are Active (HOLA) is a newly developed intervention that uses a community health worker (CHW) to lead a health promotion program in order to prevent common mental disorders among at-risk older Latinos. This pilot study tests the feasibility and acceptability of delivering HOLA to older, at-risk Latinos.

**Methods/Design:**

HOLA is a multi-component, health promotion intervention funded by the National Institute of Mental Health (NIMH). This prevention approach will be tested against a *fotonovela*, an enhanced psychoeducation control condition, in a sample of Latino elderly with minor or subthreshold depression or anxiety. A total of 60 older Latinos (aged 60+) will be randomized to receive HOLA or the *fotonovela*. The primary outcomes of interest are recruitment, adherence, retention, and acceptability. Data will also be collected on: preemption of incident and recurrent major depression, generalized anxiety, and social phobia; reduction in depression and anxiety symptom severity; physical functioning; sedentary behaviors; social engagement; and self-efficacy.

**Discussion:**

The results of this study could have implications for other high-risk, highly disadvantaged populations. The development of a health promotion intervention designed to prevent common mental disorders could be a means of addressing multiple disparities (for example, mental health outcomes, mental health service use, stigma) among racial/ethnic minority elderly.

**ClinicalTrials.gov Identifier:**

NCT02371954. Date of registration: 21 January 2015.

## Background

Latinos are the largest and fastest growing segment of the older adult population [1]. High prevalence of common mental disorders (depression and anxiety) combined with mental health service use disparities make older Latinos a high-risk population for whom scalable preventive interventions could have great public health impact [2, 3]. In addition, older Latinos are more sedentary and are disproportionately affected by diabetes and obesity compared to their non-Latino peers [4, 5]. Cultural beliefs about the causes of mental illness and stigma associated with help seeking may further contribute to Latinos’ limited access and low utilization of mental health care [6–8]. These factors underscore the need to create culturally appropriate preventive interventions for older Latinos. Effective approaches to this challenge are likely to involve using non-traditional means (for example, community health workers, health promotion) that are acceptable and scalable.

The Institute of Medicine [9] states that prevention interventions are those that are conducted before subjects meet the formal criteria for a mental disorder. There are three types of prevention interventions: (a) universal prevention is aimed at the general population, regardless of level of risk; (b) selective prevention is aimed at high-risk groups who have not yet developed a mental disorder (such as experiencing a stressful life event, including a change in health status that may limit independence, divorce, losing a family member through death, caring for an ill family member, or unemployment); and (c) indicated prevention is aimed at individuals who have some symptoms of a mental disorder but do not meet diagnostic criteria. Focusing on those at highest risk, particularly the elderly who have subthreshold symptoms (“indicated” prevention), is the most efficient way to address mental illness prevention in later life [10].

Minor and subthreshold depression and anxiety are highly prevalent in older adults and interfere with functioning similar to disorders meeting full Diagnostic and Statistical Manual (DSM) criteria [11–18]. Despite its label, minor (or subthreshold) depression carries a significant public health burden. Minor depression is a debilitating disorder that is associated with psychological suffering and significant decrements in health [15]. It is also a strong risk factor for major depression, and might increase the risk of death in older individuals [11, 12, 15]. Similarly, subthreshold anxiety is associated with significant impairment in social, family, and occupational functioning, poor perceived emotional and physical health and well-being, low satisfaction with daily life, and an increased risk for developing an anxiety disorder [13, 14, 16–18].

Although prevalence rates are high and negative health consequences are numerous and incapacitating, treatment options for minor depression and subthreshold anxiety are limited. Available treatments are difficult to access and only partially satisfactory in reducing symptom burden, sustaining remission, and averting years lived with disability [19–21]. While antidepressant medications are the most widely used modality for treating prevalent cases of major depression and anxiety, their use in subthreshold depression and anxiety is not supported by evidence of effectiveness and is more likely to be associated with adverse effects in older adults [22]. Additionally, older Latinos are less likely to find antidepressant medication acceptable than non-Latino Whites [23]. Thus, psychosocial strategies are more suitable preventive interventions.

Health promotion interventions are behaviorally activating, reduce vulnerability factors, and may be more desirable for reasons of safety and patient preference. Studies have consistently shown that increased physical activity effectively reduces symptoms of depression and anxiety as well as physical functioning risk factors in older adults [24–27]. Similarly, increasing pleasant events has been shown to be an effective intervention for geriatric depression [28]. Social engagement and self-efficacy have also been shown to be important protective factors in preventing late life depression and anxiety [29, 30]. Thus, there may be a synergy between increased physical activity and pleasant events. In this context, a health promotion intervention such as Happy Older Latinos are Active (HOLA) may be particularly promising since it combines these two strategies to reduce vulnerability factors and enhance protective factors associated with late life depression and anxiety. This makes physical activity interventions appropriate for older Latinos, who often have a combination of physical and mental health problems, by minimizing the stigma sometimes associated with antidepressant medication and talk therapy [4, 5, 8].

The number of geriatric mental health specialists is inadequate to meet the current and future needs of the Latino elderly [31–33]. One promising approach is the use of lay community health workers (CHWs) to deliver simple, scalable interventions. CHWs are lay community members who work almost exclusively in community settings and effectively connect consumers to providers in order to promote health and prevent diseases among groups that have traditionally lacked access to adequate care [34]. They have long been accepted as important conduits of health information, particularly in Latin America and as part of health-promotion efforts with diverse populations. CHWs are assumed to be effective because they are part of the communities in which they work — ethnically, socioeconomically, and experientially. They possess an intimate understanding of community social networks, strengths, and health needs; communicate in a similar language; and recognize and incorporate culture to promote health and health outcomes [35, 36]. The use of CHWs has emerged as a strategy to reduce or eliminate health disparities and is an important means of task shifting to enable more efficient utilization of scarce mental health resources [36, 37].

The combination of high exposure to risk factors (comorbid physical and mental health conditions) and disparities in access and engagement in mental health services attests to the greater illness burden of common mental disorders experienced by older Latinos. There is a compelling need for interventions that promote prevention of common mental disorders among Latino elders and are specifically designed to address their views concerning the causes of mental illness, mental health treatment, and stigma towards traditional mental health services.

The three specific aims of this pilot study are:

1. To develop and refine Happy Older Latinos are Active (HOLA), a health promotion intervention, led by a CHW, to address prevention of common mental disorders in a group of older Latinos with minor and subthreshold depression and anxiety.
2. To evaluate the feasibility and potential effectiveness of the newly refined HOLA compared to an enhanced psychoeducation condition with respect to: (a) depression and anxiety prevention and (b) depression and anxiety severity.
3. To evaluate the feasibility and potential effectiveness of HOLA compared to enhanced psychoeducation with respect to: (a) physical functioning; (b) psychosocial functioning (social engagement and self-efficacy); and (c) sleep to manage anxiety/depression.

## Methods

All study methods and protocols were approved by the Internal Review Board (IRB) of the University of Miami (UM; IRB ID: 20140607).

### Participants

The pilot study will randomly assign 60 older (60+) Latino participants with minor or subthreshold depression or anxiety to the HOLA intervention (n = 30) or enhanced psychoeducation (n = 30). The sample size is sufficient to provide meaningful data on the feasibility and the other outcome measures of interest. Additionally, this sample size is consistent with other pilot studies of physical activity interventions [38, 39]. Informed consent will be obtained from each participant. For a detailed description of the inclusion/exclusion criteria, see Table 1.

**Table 1:** Inclusion/exclusion criteria

Inclusion criteria	Exclusion criteria
• Self-identify as Latino; • Age 60+; • Have minor depression as defined by a primary DSM-IV Axis I diagnosis of minor depressive disorder or subthreshold depression defined as a score ≥ 3 on the Patient Health Questionnaire (PHQ-2), OR subthreshold anxiety as defined as a score ≥ 3 on the Generalized Anxiety Disorder-2 scale (GAD-2); • Absent of episodes of major depression and anxiety disorders for past 12 months; • Volunteer informed consent; • Have medical clearance for participation in an exercise program by a physician, physician’s assistant, or nurse practitioner; • Agree not to use antidepressant medication (since these subjects could have a partially treated episode of major depression or anxiety and thus would be inappropriate for a prevention trial); • Expect to stay in Miami for the next 12 months	• Currently residing in a nursing or group home; • Have a terminal physical illness expected to result in their death within one year; • Have a diagnosis of dementia, comorbid diagnosis of dementia, or significant cognitive impairment; • Presence of any Axis I psychiatric disorder or substance abuse during preceding 12 months; • History of psychiatric disorders other than non-psychotic unipolar major depression or anxiety disorder; • High suicide risk; intent or plan to attempt suicide in the near future; • Taking cognitive enhancing medication or psychotropic medications (e.g., antidepressants); • Unable to complete 10-meter walk test in less than 23 seconds average; • Heart rate exceeds 170 beats per minute OR systolic blood pressure exceeds 180 during 10-meter walk test; • Chest or leg pain, dyspnea, dizziness, feeling faint, or other significant symptoms while completing 10-meter walk test; • Have contraindications to exercise outlined in the American College of Sports Medicine standards.

### Recruitment

A community-based recruitment strategy employing locations that older Latinos are likely to frequent (such as churches, laundromats, libraries, and hair salons) will be used. It is the mission of the Community Engagement Group to help facilitate links between researchers at UM and the community. The recruitment efforts will be led by the CHW, who will work with the community advisory board and the study team to devise recruitment strategies that are most appropriate. To the extent possible these would be informed by already successful attempts in this community. Examples include targeting high-volume areas (such as laundromats, hair salons, and flea markets) and using ads in the local Latino press (such as the newspaper and radio). In addition, local housing authorities, senior centers, and community health centers will be informed of the study and provided with promotional materials. The Miami-Dade County Parks and Recreation Department puts on regularly scheduled health fairs in which residents are provided with free health screens. The principal investigator (PI) and CHWs will be present at these events to ensure that every older adult who comes in for a health screening will be screened for depression and anxiety. At the same time, the UM Public Relations (PR) department will be informed and notified of the ongoing research. The PR department will be provided with the promotional materials for the study. UM sites such as those that make regular postings and newsletters will also be contacted and provided with an approved ad or communication regarding the project. The ad will also be posted in non-UM newsletters and advertisement sections.

### The interventions

**Fig. 1:**
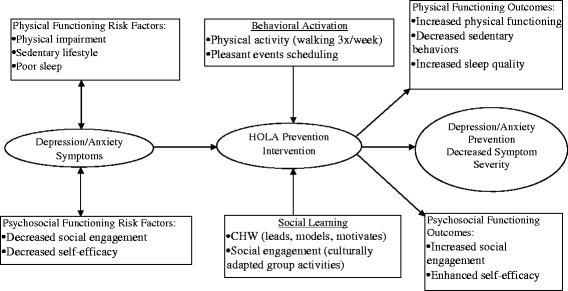
Conceptual framework

The HOLA intervention focuses primarily on indicated prevention, enrolling participants with subthreshold symptoms. The conceptual model, which forms the basis of the intervention (Fig. 1), is designed to address depression and anxiety symptoms as well as physical and psychosocial functioning. It will achieve this goal through two related mechanisms, Behavioral Activation (BA) and Social Learning Theory (SLT) [28, 40]. The intervention uses a BA [28] approach to engage in a physical activity routine as well as a pleasant events schedule to preempt incident and recurrent episodes of major depression and anxiety disorders and to reduce symptom severity. To complement this process, the constructs of observational learning, reinforcement, and enhanced self-efficacy postulated in SLT [40] are used. The relationship between the participants and the CHW capitalizes on the personal relationship to motivate, model, and maintain health behavior change. The CHW holds the individuals accountable and individuals hold themselves accountable to the group, providing extra motivation to engage in the intervention.

HOLA is a multi-component, health promotion intervention designed to prevent depression and anxiety in at-risk elderly Latinos. This prevention approach will be tested against a *fotonovela*, an enhanced psychoeducation control condition in a sample of Latino elderly with minor or subthreshold depression or anxiety. As described below, HOLA builds on prior work research [27] using physical activity to treat major depression in older adults.

#### Intervention description: content and structure of HOLA

The first component of HOLA is a social and physical activation session. Prior to beginning the group exercise phase, each participant will meet individually with a CHW for the physical and social activation session. This session will last 30 minutes. The purpose of this session is to (a) educate potential participants about the goals of the intervention; (b) motivate participants to engage in physical activity; (c) increase participants’ social activities; (d) identify potential obstacles that may interfere with meeting the demands of the intervention; and (e) brainstorm ways to overcome these obstacles. Participants will meet individually with the CHW for 30 minutes after week 8 to discuss progress of physical and social activity goals.

The second component is a moderately intense, group walk, led by a CHW for 45 minutes, three times a week, for 16 weeks. Moderate intensity is defined as a rating between 11 and 13 on the Borg Scale of Perceived Exertion [41]. Walks will be conducted with a group of six participants. Each walk will begin with 10 minutes of stretching and warm up. Then, participants will walk for 30 minutes. The walk will conclude with 5 minutes of stretching and cool down. Blumenthal and colleagues [27] showed that this intensity and amount of physical exercise is associated with greater remission of major depression in older adults. It is hypothesized that this amount and frequency will also be effective in the prevention of major depression and anxiety disorders in older Latinos with subthreshold symptoms. The groups will be mixed gender and will include bilingual and monolingual Spanish-speaking participants.

The third component consists of scheduling pleasant events. During the cool down phase of each walking session, the CHW will ask each participant to identify a pleasant event that they intend to do with another person before the next meeting. Individuals may choose to do this activity with another member of the group, with family, or with friends outside the group. The CHW will be trained to problem solve (for example, brainstorm) if a member of the group cannot think of a pleasant event. Subsequent sessions will start with participants reporting on how effectively they implemented their pleasant event plan while the CHW and the group provide positive reinforcement and feedback. This component provides a means to generalize the intervention into the participants’ everyday lives and relationships.

Mood ratings will be taken directly before and after each walk so participants can see, in real time, changes in their moods. A pedometer will be given to each participant prior to the first group walk, and will be used to track each participant’s progress and provide motivation. The group exercise will be carried out at a centrally located park that is owned and operated by the Miami-Dade County Parks and Recreation Department and is open to the public.

#### Comparison condition: content and structure of enhanced psychoeducation through *fotonovela*

The *fotonovela* was chosen as a control condition over usual care because it is a health-related intervention in its own right and relevant to older mentally ill Latinos who may have low literacy [42]. *Fotonovelas* are booklets that use posed photographs and simple text bubbles to portray soap opera stories that convey educational messages. *Fotonovelas* differ from common educational materials in that they incorporate popular images, cultural norms, simple text, dramatic stories, and vivid pictures to raise awareness, promote health, and combat stigma [42, 43]. The *fotonovela* has not been shown to be effective in increasing mental health service use, nor has it been used as mental health treatment or as a prevention tool [43]. It, however, will help patients become aware of their condition.

Participants randomized to the enhanced psychoeducation will be given a copy of the theoretically informed and empirically grounded *fotonovela* developed by Cabassa and colleagues [44]. In addition, participants in the control condition will meet one month after they are given the *fotonovela* for an open forum to express opinions concerning the *fotonovela* as well as knowledge gained from reading the material. The PI, who is a clinical psychologist, will lead these discussion groups, which will consist of six control participants per group and last for an hour.

### Measures and analysis

#### Feasibility objective: recruitment, randomization, retention, and acceptability

As a pilot study, the randomized prevention trial will examine feasibility. Consistent with recommendations from biostatistical workgroups funded by NIH [45], this pilot study is not powered to test a hypothesis. Successful recruitment will be defined as meeting 100 % of targeted randomization (n = 60), with 20 % or less of eligible subjects refusing randomization. Randomization will be deemed adequate if the randomization scheme produces equal numbers of participants randomized to each condition. Adequate retention will be defined as 85 % or more of randomized subjects completing the post-intervention assessment. Acceptability is defined as 80 % or more of sessions attended by subjects. Data will also be collected on the proposed outcome measures to be tested in a definitive randomized controlled trial.

Study measures will be administered at baseline, end of intervention, and at 6 and 12 months post intervention. Eligible participants will be paid $25 upon completion of assessments at baseline, the end of the intervention, at 6, and at 12 months post intervention for a total of $100. Trained research assistants (RA) will administer all of the assessments and will be kept blind to randomized study assignment.

After the baseline assessment, participants will be randomly assigned to the HOLA intervention or to the *fotonovela* condition. Randomization will be carried out using an automated program embodying the strategies of permuted block randomization.

#### Screening

**Fig. 2:**
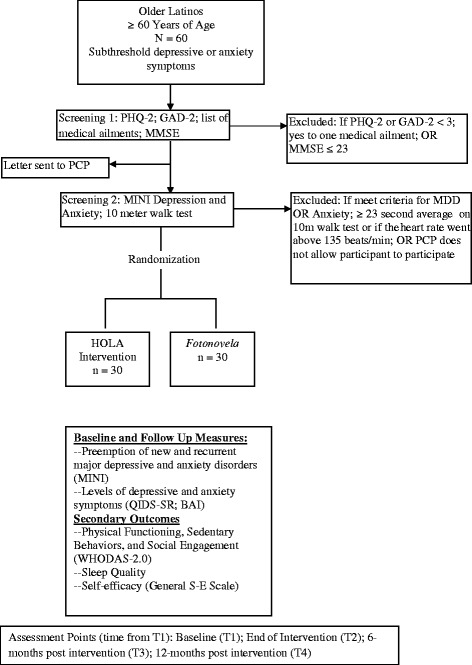
Flow chart of screening and assessment

A two-step screening process will be used to identify potential participants (Fig. 2). The first step includes the PHQ-2 [46] to screen for depression, and the GAD-2 [47] scale to screen for anxiety. The Mini Mental Status Exam (MMSE) [48] will be used to screen for dementia. Potential participants must confirm safe participation by not having an acute or severe medical illness. A letter will be sent to the subject’s primary care physician describing the study and inquiring about any conditions prohibiting participation. The second step includes screening for psychiatric diagnosis with the Mini International Neuropsychiatric Inventory (MINI) [49]. The 10-meter walk test will be used to evaluate walking ability. It has demonstrated excellent reliability when used in older adults and shows comparable results as other, longer measures [50].

#### Study measures

In the full-scale trial the primary outcomes of this study will be the preemption of new and recurrent major depressive and anxiety disorders, as measured by the MINI [49], and change in depressive and anxiety symptom severity, as measured by the Quick Inventory of Depressive Symptoms, self-report (QIDS-SR) [51] and the Beck Anxiety Inventory (BAI) [52]. Both depression and anxiety episodes will be tracked because of their frequent co-existence as common mental disorders [53], and previous prevention trials have tracked both common mental disorders [54, 55]. Secondary outcomes of the full-scale study will include change in physical functioning, social engagement, and sedentary behaviors as measured by the WHO Disability Assessment Schedule (WHODAS 2.0) [56]; change in self-efficacy as determined with the General Self-Efficacy Scale [57]; and change in sleep quality using the Pittsburgh Sleep Quality Index (PSQI) [58].

#### Analysis

Univariate analyses will be conducted to determine if randomization yielded equivalent groups by comparing demographics, background factors, key outcome variables and important covariates and potential moderators at baseline using chi-square and *t*-tests. Confidence intervals will be examined for differences between group means for all measures as a means of summarizing these analyses. If systematic differences between the two groups are found, propensity score matching will be used in statistical tests of outcomes. As a preliminary evaluation of the interventions, effect sizes will be calculated to assess for evidence of potential effectiveness. However, we accept that the study will not be powered to provide strong evidence against the null hypothesis. An intent-to-treat analysis will be conducted for the outcomes of interest, comparing the participants who were randomized to HOLA with those in the *fotonovela* condition. These analyses permit the strongest inferences to be drawn regarding the effects of physical activity and enhanced psychoeducation, but as previously discussed they may be underpowered. For a more conservative assessment, an as-treated analysis will be conducted, comparing participants randomized to and completing HOLA to those randomized to the *fotonovela*. These analyses may provide a more sensitive measure of the true effect of HOLA on reducing depression and anxiety symptom severity.

## Discussion

Many racial/ethnic minority elderly view traditional mental health services as highly stigmatizing [8, 59, 60]. Since health promotion/health behavior change interventions are culturally relevant [7] and effective in reducing risk factors [24, 29], they may be an optimal prevention strategy in the high-risk racial/ethnic minority elderly. This study will generate evidence of a potentially scalable intervention for depression and anxiety prevention in older Latinos in the midst of a rapid demographic transition. The use of a CHW delivering a health promotion intervention to prevent common mental disorders in Latino elderly is an innovative approach to reduce disease burden in a population living with high exposure to risk factors (comorbid physical and mental health conditions) and disparities in access to and engagement in mental health services [2–6]. Since increased physical activity and pleasant events are each effective in treating geriatric depression and anxiety [25–29], this study will determine whether an intervention that combines these two approaches will be acceptable, feasible, safe, and effective in preventing common mental disorders in older Latinos. By shifting the focus to prevention, and adding a CHW, two physical and social activation sessions, pleasant events scheduling, mood ratings, and pedometers, HOLA is an innovative and self-motivating intervention that makes efficient use of limited resources.

The integrated (mental and physical health) nature of this intervention enhances the innovation and potential clinical impact. In addition, the results of this study could also have implications for other high-risk, highly disadvantaged populations. Older African Americans have low rates of mental health service use [3], experience high stigma [59], and have high rates of co-morbidities [61]. Older Asian Americans also report high stigma [60] and are more likely to express distress through somatic symptoms rather than psychological terms. [62]. All of these groups could potentially benefit from the proposed health promotion intervention in the service of preventing common and disabling mental disorders. The development of a health promotion intervention designed to prevent common mental disorders could be a means of addressing multiple disparities (for example, mental health outcomes, mental health service use, stigma) among racial/ethnic minority elderly people.

### Trial status

Study recruitment, assessment, and intervention delivery activities are ongoing at the time of submission.

## Abbreviations

BA: Behavioral Activation
BAI: Beck Anxiety Inventory
CHW: community health worker
DSM: Diagnostic and Statistical Manual
GAD-2: 2-item Generalized Anxiety Disorder scale
General S-E Scale: General Self-Efficacy Scale
HOLA: Happy Older Latinos are Active
IRB: Internal Review Board
MDD: Major Depressive Disorder
MINI: Mini International Neuropsychiatric Inventory
MMSE: Mini Mental Status Exam
NIH: National Institute of Health
NIMH: National Institute of Mental Health
PCP: primary care physician
PHQ-2: 2 item Patient Health Questionnaire
PI: principal investigator
PR: Public Relations
PSQI: Pittsburgh Sleep Quality Index
QIDS-SR: Quick Inventory of Depressive Symptoms, self-report
RA: research assistant
SLT: Social Learning Theory
T1: Time 1
T2: Time 2
T3: Time 3
T4: Time 4
UM: University of Miami
WHODAS 2.0: World Health Organization Disability Assessment Schedule, version 2.0
